# Conserved and Divergent Features of pH Sensing in Major Fungal Pathogens

**DOI:** 10.1007/s40588-023-00195-5

**Published:** 2023-07-28

**Authors:** Shadab Farhadi Cheshmeh Morvari, Bethany L. McCann, Elaine M. Bignell

**Affiliations:** 1 Manchester Fungal Infection Group, University of Manchester, Grafton Street, Manchester, M13 9NT UK; 2 Medical Research Council Centre for Medical Mycology at The University of Exeter, Stocker Road, Exeter, EX4 4QD UK

**Keywords:** *Aspergillus*, *Candida*, *Cryptococcus*, pH signalling, Adaptation, Virulence

## Abstract

**Purpose of Review:**

For human fungal pathogens, sensory perception of extracellular pH is essential for colonisation of mammalian tissues and immune evasion. The molecular complexes that perceive and transmit the fungal pH signal are membrane-proximal and essential for virulence and are therefore of interest as novel antifungal drug targets. Intriguingly, the sensory machinery has evolved divergently in different fungal pathogens, yet spatial co-ordination of cellular components is conserved.

**Recent Findings:**

The recent discovery of a novel pH sensor in the basidiomycete pathogen *Cryptococcus neformans* highlights that, although the molecular conservation of fungal pH sensors is evolutionarily restricted, their subcellular localisation and coupling to essential components of the cellular ESCRT machinery are consistent features of the cellular pH sensing and adaptation mechanism. In both basidiomycetes and ascomycetes, the lipid composition of the plasma membrane to which pH sensing complexes are localised appears to have pivotal functional importance. Endocytosis of pH-sensing complexes occurs in multiple fungal species, but its relevance for signal transduction appears not to be universal.

**Summary:**

Our overview of current understanding highlights conserved and divergent mechanisms of the pH sensing machinery in model and pathogenic fungal species, as well as important unanswered questions that must be addressed to inform the future study of such sensing mechanisms and to devise therapeutic strategies for manipulating them.

## Introduction

In fungi the ambient pH of the extracellular niche governs the expression and functionality of multiple secreted and cell surface–associated gene products that must be nimbly moderated to maintain nutrient acquisition, cell wall homeostasis and cation tolerance [1–3]. In the mammalian host, fungi are often exposed to a wide range of pH values that vary according to the tissue niche and inflammatory milieu. Therefore, understanding the functionality of mechanisms that promote versatility under pH flux is crucial for understanding how fungi are pathogenic and for informing improved disease control, particularly in the invasive disease-causing species recently classified by the World Health Organisation as being of critical priority; *Aspergillus*, *Candida* and *Cryptococcus* species [4, 5•].

Fungal pH adaptation, including in pathogens, relies upon highly conserved, mostly fungus-specific molecular mechanisms that converge upon pH-responsive transcription factors named PacC in filamentous fungi [6–8] or Rim101 in yeasts [9]. Intriguingly, the sensory machinery, that functions upstream of transcription factor activation, has evolved divergently in different fungal pathogens, yet spatial co-ordination of components is conserved. Relative to founding mechanistic studies conducted in the model ascomycetes *Aspergillus nidulans* and *Saccharomyces cerevisiae*, we here compare the pH sensing machinery of different fungal pathogens, reviewing recent research and identifying interesting new questions that are raised. The resultant overview of current understanding is intended to inform future study of the sensing mechanisms and therapeutic strategies for manipulating them.

**Fig. 1:**
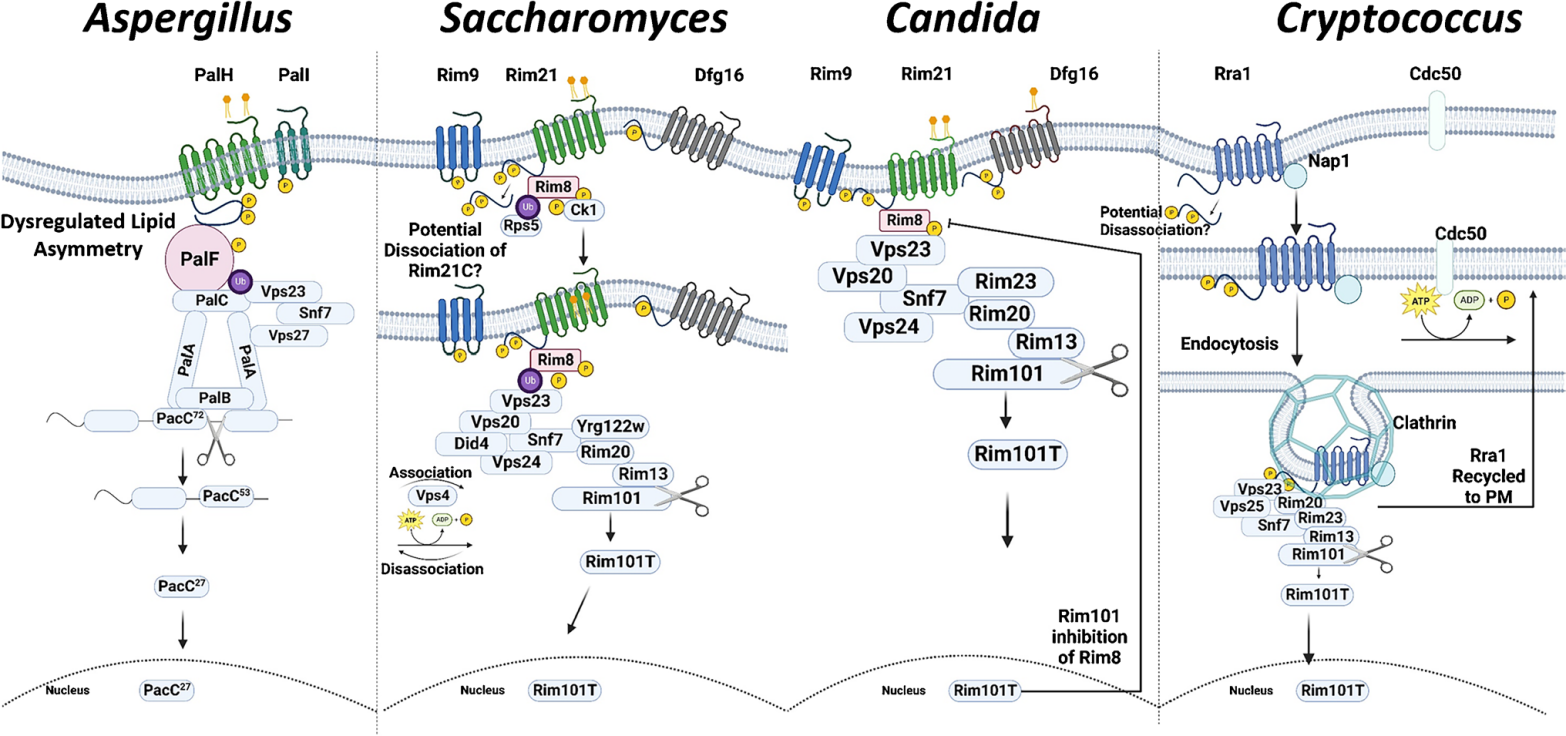
Activation of pH-regulated transcription factor PacC/Rim101 in response to alkalinisation. Adaptation to host-imposed environmental conditions, including wide ranges of pH, is a crucial virulence determinant of human fungal pathogens, such as those recently classified by the WHO as of critical concern: *Aspergillus fumigatus*, *Candida albicans*, *Candida auris* and *Cryptococcus neofomans* (5). The machinery and mechanisms required for pH sensing and adaptation have been widely studied in the model organisms, *S. cerevisiae* and *A. nidulans*. Although all pathogens must adapt to a wide pH range in order to colonise hosts, there are a number of divergent mechanisms, particularly those required by the basidiomycete *C. neoformans*; all pathogens, however, require a 7TMD putative sensor: *An/Af*PalH (*Aspergilli*), *Sc/Ca*Rim21 (*Saccharomyces* and *Candida*) or *Cn*Rra1 (*Cryptococcus*), which (except *Cn*Rra1) complex, forms a complex with a number of other proteins required for proteolytic activation of the pH-responsive transcription factors, *An/Af*PacC *(Aspergilli*) or Rim101 *(Sc*, *Ca* and *Cn*)*.* Plasma membrane localisation of this sensor is aided by *An/Af*PalI and *Sc/Ca*Rim9, a 3/4 TMD protein. In yeasts such as *Saccharomyces* and *Candida*; an additional 7TMD protein which governs the cellular level and PM localisation of *Sc/Ca*Rim21 is required for signalling but does not act as a sensor of extracellular pH; *Sc/Ca*Dfg16. The final component of this complex, *An/Af*PalF/*Sc/Ca*Rim8, is an arrestin-like protein that interacts with the C-terminal tail of *An/Af*PalH and *Sc/Ca*Rim21. Homologues of Rim8, Rim9 and Dfg16 are absent in *C. neoformans*, membrane localisation is aided by *Cn*Nap1. Upon environmental alkalinisation, *An/Af*PalH and *Sc/Ca*Rim21 and *Sc*Dfg16 become both phosphorylated and N-glycosylated, and the loss of glycosylation of *Sc*Rim21 alters localisation compared to glycosylated forms. Both phosphorylation and glycosylation are dispensable for function. *An/Af*PalH and *Sc*Rim9 is phosphorylated, but not glycosylated; this occurs in a *Sc*Dfg16-dependent manner*. Cn*Rra1 is phosphorylated at both acidic and alkaline pH, however differentially. The C-terminal tails of the pH sensors: *An/AfPalH *and *Sc/Ca*Rim21 and *Cn*Rra1 are seemingly critical for functionality; it is here that the sites required for interaction with *An/Af*PalF and *Sc/Ca*Rim8 are found. The functionality of the tail, including localisation/interaction with the PM, relies on the presence of highly charged sequences of AAs. In both *Saccharomyces* and *Cryptococcus*, dissociation of the C-terminal tail away from the PM has been visualised using epifluorescence microscopy. PTM of *An/Af*PalF/*Sc*Rim8 is essential for the recruitment of downstream-acting components, including the ESCRT complexes, to punctate locations on the plasma membrane. In *Candida*, phosphorylation of *Ca*Rim8 occurs in a CK1-dependent manner; in strains lacking CK1, *Ca*Rim101 is constitutively activated. Under acidic conditions, *Ca*Rim8 is hypo-phosphorylated and constitutively localised, in complex with *Ca*Rim21 to the vacuole; however, under alkaline conditions, *Ca*Rim8 is hyper-phosphorylated and localised at the PM. Ubiquitination of the C-terminus of *An/Af*PalF is crucial and in *Sc*Rim8 is ubiquitinated in an Rsp5-dependent manner; through the interaction of Rsp5 with the PXY motif of *Sc*Rim8, this is independent of *Sc*Rim21 or *Sc*Dfg16 but dependent on expression of the ESCRT component Vps23. Ubiquitination of *Ca*Rim8 has not been detected. in *Aspergilli*, the PTM of *Af*PalF is dependent on *Af*PalH; a fusion of a ubiquitin moiety to *An/Af*PalF is able to circumvent the need for *An/Af*PalH for proteolytic activation of *An/Af*PacC, further confirming how critical PTM of *An/Af*PalF/*Sc*Rim8 is for pH signalling. Ubiquitination of *Af*PalF/*Sc*Rim8 results in the recruitment of a number of ESCRT components: first, the conserved Vps23, this subsequently leads to the recruitment of Snf7, other ESCRT machinery, the Vps32 interacting *An/Af*PalC and *Sc/Ca*Rim23 and *An/Af*PalA and *Sc/Ca*Rim20 and the cysteine protease responsible for cleavage of *An/Af*PacC and *Sc/Ca*Rim101 and *An/Af*PalB and *Sc/CA*Rim13. As ubiquitination of *Ca*Rim8 has yet to be detected, the mechanism of recruitment of downstream components is not well characterised; however, all components are conserved as in *Saccharomyces.* As Rim8 is absent in *Cn* the mechanism of recruitment of ESCRT and downstream acting components and complexes is thought to involve clathrin-mediated endocytosis of *Cn*Rra1. Endocytosis of membrane components is dispensable for function in *Saccharomyces* and *A. nidulans*; however, it does have a role in recycling inactive *An/Af*PalH and *Sc/Ca*Rim21 back to the PM. A negative feedback loop that governs pathway activity, including proteolytic activation of *Ca*Rim101, has been identified in *Candida*, where activated *Ca*Rim101 negatively regulates *Ca*Rim8

A comparison of pH sensing in *Aspergillus nidulans (An), Aspergillus fumigatus (Af)*, *Saccharomyces cerevisiae* (*Sc*), *Candida albicans* (*Ca*) and *Cryptococcus neoformans* (*Cn*) is provided in Table 1 and Fig. 1.

**Table 1:** pH sensing in model and pathogenic fungi

Genus	pH Sensing Component	Role	Conditions activating pH signalling	Importance of C-terminus for function	Dissociation of the C-terminus	PTM*	Endocytosis	Required for virulence	ESCRT machinery required for signalling	pH-responsive transcription factor	References
*Aspergillus*	PalH	Presumed pH sensor	Alkaline pH, stoichiometrically equivalent expression of PalH and PalI	Interaction with PalF	Unknown	N-glycosylation and phosphorylation. Non-essential	Not essential for function, PalH recycled to PM	Yes	Vps23, Vps36, Vps20, Vps32/Snf7	PacC	(3, 6, 7, 19, 25, 32, 34, 36, 38, 43)
	PalI	Aids PM localisation				Unknown	Unknown	Unknown			
	PalF	Aids PM localisation Transduces pH signal Recruits ESCRT machinery				Ubiquitinated and phosphorylated Essential for function	Unknown	Unknown			
*Saccharomyces*	Rim21	Presumed pH sensor	Neutral-alkaline pH	Interaction with Rim8, interaction with, and localisation to, PM	C-terminal dissociated	N-glycosylation and phosphorylation. Non-essential	Not essential for function, Rim21 recycled to PM	N/A	Vps23, Vps32/Snf7, Vps25, Vps36, Snf7, Vps20, Did4	Rim101	(9, 13, 29, 40, 41, 43, 44)
	Dfg16	Aids PM localisation of Rim21				N-glycosylation and phosphorylation but non-essential for function					
	Rim9	Aids PM localisation of Rim21				phosphorylation. Non-essential					
	Rim8	Aids PM localisation of Rim21 Transduces pH signal Recruits ESCRT machinery			No	Ubiquitination, essential for function, Rsp5 dependent					
*Candida*	Rim21	Presumed pH sensor	Neutral-alkaline pH	Unknown	No	N-glycosylation and phosphorylation. Non-essential	Unknown, presumed as *S. cerevisiae*	Yes	Vps28, Vps36, Vps22, Vps20, Snf7	Rim101	(11, 14, 15, 30, 33, 42, 45)
	Dfg16	Aids PM localisation of Rim21		Unknown		N-glycosylation and phosphorylation. Non-essential					
	Rim9	Aids PM localisation of Rim21				Putative phosphorylation					
	Rim8	Aids PM localisation of Rim21 Transduces pH signal Recruits ESCRT machinery				Hyper-phosphorylation, Ck1-dependent Essential for function					
*Cryptococcus*	Rra1	Putative pH sensor	Neutral-alkaline ph C-terminus required for localisation to PM. Sre1 regulated PM localisation	Interaction with, and localisation to, PM	Yes	C-terminus differentially phosphorylated at acidic or alkaline pH, not essential for function	Yes, clathrin-mediated Essential for function Recycled back to PM following activation of Rim101	Yes	Vps23, Vps25, Snf7	Rim101	(2, 12, 17, 37, 45–48)

* Post-translational modification

## Fungal pH Sensors

The likely fungal pH sensors are seven-transmembrane proteins named PalH/Rim21, Dfg16 and Rra1, whose integrity is critical for activation of PacC/Rim101 signalling [9–11, 12•].

In the model ascomycete *A. nidulans*, the 760 amino acid (AA) *An*PalH has a periplasmic N-terminal moiety, seven hydrophobic membrane-spanning domains and a long hydrophilic cytosolic terminus [10]. Negrete-Urtasun et al. (1999) confirmed that at alkaline ambient pH, the plasma membrane (PM) spanning *An*PalH is required for *An*PacC processing [10]. In *A. nidulans* and *Aspergillus fumigatus*, null mutants of *An/af*PacC exhibit morphological defects, alkaline and cation sensitivity and attenuation of virulence in murine models of invasive lung infection [6, 7].

In *S. cerevisiae*, transient degradation of *Sc*Rim21 abolished pH signalling by suppressing proteolytic activation of *Sc*Rim101. Similar to *An*PalH, the predicted *Sc*Rim21 (590 AA) structure consists of seven transmembrane domains with an extracellular N-terminus and a cytosolic C-terminus [13].

Deletion of *RIM21*, encoding the (529 AA) *Ca*Rim21 in *C. albicans*, revealed the loss of function phenotype including alkaline and cation sensitivity. Additionally, the loss of *RIM21* resulted in an inability to transition from yeast to hyphae, a virulence trait goverened by *Ca*Rim101, suggesting loss of *Ca*Rim101 activation in the absence of *Ca*Rim21 [14]. In both *S. cerevisiae* and *C. albicans*, null mutants of Rim21 exhibit defects in alkaline growth and sporulation [9].

*S. cerevisiae* and *C. albicans* express a second plasma membrane (PM)-associated 7TMD protein, required for Rim signalling, Dfg16. In both species, like Rim21, Dfg16 is required for the yeast to hyphal switch under alkaline conditions. It has been speculated that Dfg16 and Rim21 act as two components of a heterodimeric receptor [15] although direct proof of this hypothesis is difficult to attain due to the poor tractability of structural studies, a problem that might be soon overcome by the advent of higher throughput CryoEM studies [16•].

There are no homologues, in *Cryptococcus neoformans*, having sequence similarity to *An*/*Af*PalH and *Sc*/*Ca*Rim21. or *Sc/Ca*Dfg16. However, *Cn*Rra1, a 7TMD protein, was identified in 2015, as being the most upstream component required for both the proteolytic processing and nuclear localisation of *Cn*Rim101. Like null mutants of *Af*PalH and *Sc/Ca*Rim21 in the ascomycetes, null mutants of *Cn*Rra1 suffer alkaline and cation tolerance defects [12•, 17].

## Other Membrane-Proximal pH-Sensing Components

In *S. cerevisiae*, *C. albicans* and *A. nidulans*, an arrestin-like protein *An*PalF or *Sc/Ca*Rim8 plays an integral role in pH sensing. There is no identified homologue of PalF/Rim8 in *Cryptococci* [17].

*An*PalF interacts with two regions of the cytoplasmic terminus of *An*PalH [18]. This interaction is conserved in *A. fumigatus*, and *S. cerevisiae*, confirmed in both instances by yeast two-hybrid analyses between *Af*PalF and *Af*PalH and *Sc*Rim8 and *Sc*Rim21 respectively [19, 20].

In the absence of *An*PalH, *An*PalF does not become ubiquitinated, a critical, pH-dependent post-translational modification required for the recruitment and engagement of downstream components of the pH adaptation mechanism [21]. Covalent attachment of a single ubiquitin moiety to the *An*PalF C-terminus (PalF-Ub) in the *An*PalH null background bypasses the requirement for *An*PalH to promote proteolytic activation of *An*PacC [22–26].

*Sc*Rim8 constitutively interacts with *Sc*Rim21 through its arrestin domain(s) and is ubiquitinated at its C-terminus by the *Sc*Rsp5 ubiquitin ligase through interaction with the PXY motif located at the C-terminus of *Sc*Rim8 [27, 28]. *Sc*Rim8 ubiquitination is critical for the binding of *Sc*Rim8 to *Sc*Vps23 (ESCRT-I) in a pH-dependent manner; however, ubiquitination does not occur in a pH-regulated manner [28]. Moreover, via immunoblot analysis, it has been shown that *Sc*Rim8 ubiquitination is not dependent on *Sc*Rim21 or *Sc*Dfg16 but is dependent on the expression of *Sc*Vps23 [28]. Thus, *Sc*Rim8 ubiquitination likely regulates pH signalling by recruiting downstream molecules to the plasma membrane [29].

*Ca*Rim8 is also subject to pH-dependent post-translational modification, becoming hyper-phosphorylated in response to extracellular alkalinisation [30•]. Mutants lacking either *Ca*Rim21, *Ca*Dfg16 or *Ca*Rim9 remain able to hypo-phosphorylate *Ca*Rim8 suggesting that phosphorylation is not dependent on the presence of these proteins. At acidic pH, hypo-phosphorylated *Ca*Rim8 interacts with non-phosphorylated *Ca*Rim21 and is constitutively trafficked to the vacuole, thereby moderating the functionality of pH adaptation via re-localisation of essential pH sensing components [28, 30•]. Although the level of phosphorylation of *Ca*Rim8 is pH-dependent, phosphorylation occurs in a casein kinase 1 (CK1)-dependent manner under both acidic and alkaline pH conditions at a Ser/Thr-rich region but requires localisation of *Ca*Rim8 at the PM [30•]. In mutant strains lacking *ck1*, *Ca*Rim8 is phospho-deficient, and *Ca*Rim101 is constitutively proteolysed under acidic conditions. These findings suggest a role for *Ca*Rim8 phosphorylation in providing a means to inhibit proteolytic activation of *Ca*Rim101 by maintaining *Ca*Rim8 phosphorylation below the required threshold for pathway activity at acidic pH acidic [31]. Unlike *Sc*Rim8 and *An*PalH, *Ca*Rim8 has not been found to be ubiquitinated, despite the existence of both a cognate lysine in close proximity to the PXY motif and *Ca*Rps5 homologues [30•]. The absence of ubiquitination of *Ca*Rim8 may be explained by the existence of divergent mechanisms for the recruitment of ESCRT components required for proteolytic activation of Rim101 or, more trivially, by the occurrence of more transient post-translational modification (PTM) of *Ca*Rim8 [30•].

In *S. cerevisiae*, *C. albicans* and *A. nidulans*, Rim9/PalI is a putative transmembrane protein functioning upstream of Rim101/PacC [32]. There is no Rim9/PalI homologue in the *Cryptococcus* species. *An*PalI is homologous with *Sc*Rim9 and based on hydrophilicity is also predicted to be a membrane-spanning protein [32, 33]. The deletion of *AnpalI*, in *A. nidulans*, leads to partial loss-of-function phenotypes under alkaline pH, with significantly diminished levels of processed *An*PacC [3, 10, 34]. Thus, based on both phenotypic and *An*PacC processing data, it can be concluded that *An*PalI contributes to pH signalling but is somewhat dispensable. The deletion of *Carim9* significantly impacts the cellular responses to alkaline pH, via a complete loss of proteolytic activation of *Ca*Rim101 [14].

The cellular content of *Sc*Dfg16 is reduced in the absence of *Sc*Rim9 but not in the absence of *Sc*Rim21 [13, 35].

Transcription of Rim21, Dfg16 and Rim9 in neither *S. cerevisiae* nor *C. albicans* is pH-regulated. However, in both species following neutral-alkaline pH shifts which result in the proteolytic activation of Rim101, transcription of Rim8 is rapidly reduced. Reduction in *Sc/Ca*Rim8 transcription therefore results in a negative feedback loop that acts to prevent further transduction of alkaline pH response signals [30•].

## pH-Sensing Protein Complexes: Assembly and Subcellular Localisation

Obara et al. (2012) investigated the localisation, physical interaction and interdependency of pH sensing proteins in *S. cerevisiae*, proposing that *Sc*Rim21 functions as a pH sensor, with *Sc*Dfg16 and *Sc*Rim9 being required to maintain the stability/total cellular quantity of *Sc*Rim21, presumably by facilitating its PM delivery and localisation [13].

GFP-tagged *Sc*Rim21, *Sc*Dfg16 and *Sc*Rim9 proteins were primarily detected at the PM, with some detection at intracellular membranes. Interestingly, localisation of *Sc*Rim21, *Sc*Dfg16 and *Sc*Rim9 was significantly altered in mutants lacking two out of three components, where PM localisation of *Sc*Rim21 is undetectable in the *Scrim9* or Sc*dfg16* null isolates. Interaction between *Sc*Rim21, *Sc*Dfg16 and *Sc*Rim9 was confirmed using co-immunoprecipitation pull-down assays [13].

In *A. nidulans* subcellular localisation studies carried out at acidic pH of *An*PalH-GFP, expressed under the control of an over-expressing promoter *alcA*^*p*^, confirmed that *An*PalH localises at the PM, but it predominantly accumulates in cytosolic compartments [21, 34]. Given the likelihood of aberrant localisation when the pH sensor is expressed to physiological excess, a subsequent analysis via co-overexpression of both *An*PalH-GFP and *An*PalI-HA_3_, at stoichiometrically equivalent levels, resulted in the predominant localisation of *An*PalH at the PM. Similar to the situation in *S. cerevisiae*, therefore, *An*PalI likely has a role in assisting localisation of *An*PalH at the PM [21, 34]. *An*PalF is also involved in assisting *An*PalH PM localisation, as co-overexpression of *An*PalH-GFP and *An*PalF resulted in the localisation of *An*PalH at the PM [21]. These findings also suggest that under acidic pH, *An*PalH, *An*PalI and *An*PalF are interdependent components of a complex that is required for the correct localisation of the pH sensing machinery. Maintenance of this complex at the PM has not been investigated, as construction of the required strain, which co-overexpresses *An*PalH, *An*PalI and *An*PalF at stoichiometrically equivalent levels, would likely deplete components of the downstream ESCRT machinery, adversely affecting *An*PacC activation [36]. Thus, in *A. nidulans*, no subcellular localisation studies, with physiologically relevant or stoichiometric overexpression of one or more components, have been carried out at alkaline pH.

In *C. neoformans*, *CnRra1* is localised to the PM, in a manner dependent on (i) integrity of the *Cn*Rra1 C-terminus [37] and (ii) extracellular pH (2). GFP-tagged truncated *Cn*Rra1 (CnRra1-296 T-GFP), which lacks the majority of the C-terminus, but retains a highly charged region, immediately downstream to the final TMD, is functional and exhibits similar localisation patterns to the full-length version of *Cn*Rra1 at both pH 4 and pH 8 with punctate structures forming at the cell surface in lower pH conditions and an increase in endomembrane staining at pH 8. More severe truncation of the C terminus results in loss of proteolytic activation of *Cn*Rim101 presumably via mislocalisation of the protein to intracellular and “perinuclear punctate structures” at both acidic and alkaline pH [2, 12•, 37].

## Importance of Endocytosis for Fungal pH Sensing

Whilst the evidence for fungal pH sensing complexes to localise to the PM is compelling, the spatial and functional convergence of pH sensing complexes with components of the endocytic machinery might differ by fungal species.

Epifluorescence microscopy and pulldown studies indicated that the ESCRT machinery of *A. nidulans* may be recruited to punctate sites at the cytosolic side of the PM. AnVps23, the ubiquitin-binding vacuolar protein sorting (VPS), ESCRT1 component, is conserved and universally required for proteolytic activation of pH-responsive transcription factors in pathogenic fungi [2, 27]. *AnVps23* was co-immunoprecipitated exclusively with ubiquitinated *An*PalF; additionally, *An*Vps23 localised to punctate inner leaflet sites of the PM in an *An*PalF-dependent manner [38].

In *A. nidulans*, using SynA as a surrogate marker, the endocytosis of the pH signalling complex was assessed via a secretory V-SNARE internalisation assay; maintenance of SynA at the PM is indicative of inhibition of endocytosis. In endocytosis-deficient mutants, no alteration in the level of *An*PacC activation was detectable [36]. Under conditions where *An*PalH localisation to the PM is not stably maintained, i.e. in the absence of, or in strains without stoichiometrically equivalent expression of *An*PalI or *An*PalF, recycling endocytosis of *An*PalH is provoked. Under physiologically relevant levels of expression of *An*PalH, it seems that *An*PalF stabilises the PM localisation whilst also promoting the recruitment of downstream-acting pathway components [36].

As in *A. nidulans*, inhibition of endocytosis did not affect the activation of the *Sc*Rim101 pathway in *S. cerevisiae*, and the endocytosis of *Sc*Rim21 is considered to turn over stimulated *Sc*Rim21 following successful signal transduction [29]. In *Saccharomyces*, *Sc*Rim components downstream of *Sc*Rim21, accumulate at the PM in a *Sc*Rim21-dependent manner following alkaline stresses. *Sc*Snf7/*Sc*Vps32, a highly conserved and abundant component of ESCRTIII, universally required in the proteolytic processing of pH-responsive transcription factors [2, 27] localises in both the PM and the late endosome under alkaline pH; only PM localisation of *Sc*Snf7 is essential for *Sc*Rim101 signalling [29]. Co-overexpression of *Sc*Rim8 and *Sc*Vps23 results in the accumulation of both *Sc*Rim8 and recruited *Sc*Vps23 at the PM, under acidic conditions [28].

In response to environmental alkalinisation, *Cn*Rra1 localises first to endocytic vesicles, then to endomembranes such as the perinuclear endoplasmic reticulum or intracellular vesicles. *Cn*Rra1 is maintained within membranes via *Cn*Nap1 (nucleasome adaptor protein 1) [37]. Acidification of previously alkaline environments results in the recycling of *Cn*Rra1 from internal membranes to punctate PM loci; this recycling also occurs, following the successful activation of *Cn*Rim101. This endocytosis of *Cn*Rra1 is clathrin-dependent, whereby clathrin coating of *Cn*Rra1 vesicles results in the recruitment of ESCRT complexes and downstream-acting *Cn*Rim pathway components. Pitstop-2-mediated inhibition of clathrin-dependent endocytosis results in a decrease in Rim101 nuclear localisation [37]. This indicates that clathrin-mediated endocytosis of *Cn*Rra1 is essential for the activation of *Cn*Rim101.

## The C-Terminus of PalH/Rim21/*Cn*Rra1 Plays Crucial Roles in Both the Localisation and Function of the pH Sensor

The C-terminus of *Sc*Rim21 starts from amino acid 301 and ends at amino acid 533 [39]. *Sc*Rim21C is enriched in charged amino acids and interacts with the inner leaflet of the PM, Nishino and colleagues showed that GFP-*Sc*Rim21C was primarily located at the plasma membrane at acidic pH; external alkalisation resulted in the disassociation of GFP-Rim21C from PM and localisation to the cytosol and the nucleus at pH 8. Following re-acidification of the environment to pH 4.5, GFP-Rim21C localises to the PM within 5 minutes [40•]. To determine whether charged amino acid clusters located in *Sc*Rim21C are important for *Sc*Rim21 functionality, site-directed mutagenesis of *Sc*Rim21C conducted in a strain lacking the full-length *Sc*Rim21 revealed that three consecutive Glu residues (353–355) of an EEE motif were essential. In this situation, the postulated reason for the lack of *Sc*Rim101 activation is aberrant recruitment of a downstream Rim component, *Sc*Rim20.

In *A. nidulans*, the C-terminal domain of *An*PalH contains two high-affinity *An*PalF-binding sites, one directly adjacent to TM7 at residues 349 to 384, and then residues 654–760. To determine if the region between the two *An*PalF binding sites is essential for functional signalling, a strain (*AnpalH654*) was constructed where AAs 385 to 653 were substituted by a “synthetic linker consisting of a Gly-Ala pentamer”. Unlike the Δ*AnpalH* mutant, the *AnpalH654* variant was able to grow under alkaline conditions, and processing of *An*PacC was maintained, albeit slightly weaker than WT. Therefore, the region between the two identified *An*PalF binding sites is not essential for pH signalling (54). Residues 349–385 of the *An*PalH C-terminus are sufficient to interact with *An*PalF in two-hybrid assays (45). The importance of clusters of charged AAs in the C-terminal domain of *An*PalH has not been explored, and neither have there been any published studies on the localisation or functionality of C-terminal mutants of *An*PalH.

Comparison of the *Sc*Rim8 binding site of *Sc*Rim21 (residues 327–533) with the first *An*PalF binding site of *An*PalH revealed the presence of a conserved Trp-Glu-Trp motif (1). In *A. nidulans*, the Trp^349^-Glu^350^-Trp^351^ motif is located on the interface between the C-terminus of TM7 and the cytosolic terminus. The removal of E350 and W351 results in a complete loss of function phenotype upon exposure to alkaline pH, suggesting that this motif is critical for *An*PalH-*An*PalF interactions. Additionally, a mutation in a conserved Leu^368^ located within the first *An*PalF binding site of the cytosolic terminus of *An*PalH impaired binding of *An*PalF to *An*PalH [40•]. The effects of mutations in the conserved Trp-Glu-Trp motif and Leu have not been studied in *Sc*Rim21*.*

*Cn*Rra1C is enriched in arginine and lysine residues that are crucial for the PM localisation of the protein. When expressed in a Δ*Cn*Rra1 null, *Cn*Rra1-296 T-GFP complements the loss of function, through maintenance of a highly, positively charged region directly downstream to the TMD region; however, the *Cn*Rra1-273 T-GFP (2), which lacks these charged residues, is unable to localise to the PM or to overcome the loss of function phenotype. This highly charged region, therefore, is essential for localisation and functionality, although the mechanisms by which this occurs are not yet fully understood.

To date, a detailed analysis of the mechanistic and functional roles of the C-terminal domain of *Ca*Rim21 is lacking.

## Biophysical Determinants of pH Signalling Activation

The composition of lipids is different between the inner (cytoplasmic) and the outer (extracellular) membranes of the fungal PM, resulting in an asymmetric distribution of phospholipids, with negatively charged phosphatidylserine (PS) confined to the inner leaflet. Lipid asymmetry is generated and mediated by “ATP-dependent inward (flip) and outward (flop) trans-bilayer movements of lipid molecules”, catalysed by flippases and floppases, respectively.

Lipid asymmetry and proton electrochemical gradients, generated by differing proton concentrations inside (pH 7.4) and outside (pH 4.5) of the cell, are paramount for controlling PM polarisation [40•]. External alkalisation collapses the proton electrochemical gradient, resulting in depolarisation of the PM. Therefore, one hypothesis is that *Sc*Rim21 senses change in ambient alkaline pH by detecting the depolarisation status of the PM through a lipid sensing motif found in its C-terminal tail (13). Alternatively, *Sc*Rim21 may be able to sense alterations in lipid asymmetry caused by the protonophore, carbonyl cyanide m-chlorophenyl hydrazone (CCCP)-induced membrane depolarisation, suggesting that *Sc*Rim21C detects changes in lipids (PS) at the inner leaflet of the PM, triggering pathway activation (13). Consistent with both of these hypotheses Obara et al. (2012) showed using cells that do not express the PS synthase Cho1 and thus do not produce PS, or are defective in Lem3-regulated phospholipid asymmetry that the *Sc*Rim101 pathway can become constitutively activated under such conditions, even in the absence of an alkaline signal (13). PM depolarisation induced by CCCP also triggers the activation of *Sc*Rim101 in a *Sc*Rim21-dependent manner, in the absence of alkaline stress (13). The ability of *Sc*Rim21C to sense alterations in lipid asymmetry was analysed by monitoring the subcellular localisation of *Sc*Rim21C variants in cells mutated for lipid-synthesis or asymmetry. *Sc*Rim21C completely disassociated from PM in both *lem3∆* and *pdr5∆* cells. Thus, it was concluded that the cytosolic terminus of *Sc*Rim21 can sense and respond to the alterations in lipid asymmetry. *Sc*Rim21C variants lacking an ERKEE motif which is adjacent to the EEE motifs showed that the EEE motif has a crucial role in sensing or responding to changes in lipid asymmetry. The ERKEE motif, in particular the positively charged RK sequence, is required for *Sc*Rim21C association to the PM, whilst the negatively charged EEE motif is required for disassociation from the PM. It is therefore postulated that these motifs work together, forming a sensor. It is postulated that dissociation from the plasma membrane initiates recruitment of proteins acting downstream of *Sc*Rim21, via post-translational modification of *Sc*Rim8 (57). The flipping of three phospholipids: phosphatidylcholine, phosphatidylethanolamine and phosphatidylserine decreased significantly at alkaline pH compared to neutral and acidic [41]. In addition, it has been indicated that alteration in the lipid asymmetry of the PM resulted in an accumulation of the downstream cysteine protease required to cleave *Sc*Rim101, *Sc*Rim20 at the PM [29].

In addition to ergosterol homeostasis being a requirement for PM localisation of *Cn*Rra1, the normal asymmetry between leaflets maintains *Cn*Rra1 protein localisation in sterol-rich domains of the PM. This interaction likely occurs through charged AA interactions between the PM and the C-terminal domain of *Cn*Rra1. Temporary dissociation of the C-terminal domain of *Cn*Rra1 driving the endocytosis of *Cn*Rra1 from the PM as a result of lipid asymmetry highlights that regulation of the PM composition has a significant role in the activation of *Cn*Rim signalling in *Cryptococcus*. In strains lacking the regulatory subunit, cdc50 of the type IV ATPases, the flippases that govern maintenance of PM asymmetry [42], defects in growth in alkaline environments are exhibited. These mutant strains also exhibit a delay in nuclear localisation and activation of Rim101 (2). Cdc50 actively restores normal membrane asymmetry following external pH-induced dysregulation of the PM, which results in the disassociation of the C-terminal domain of *Cn*Rra1, its endocytosis and subsequent activation of Rim signalling (2). In Rim101 null mutants, because of dysregulated phospholipid maintenance of the PM, *Cn*Rra1 has a decreased ability to recycle to the PM, potentially due to changes in the ability of *Cn*Rra1 to interact with the PM.

## Conclusions

Adaptation to environmental pH is critical for the survival and proliferation of many clinically important fungi. The inability to adapt to pH flux often results in loss of fitness, virulence or viability. Such adaptations require precise governance of gene expression that is dependent upon transcription factor activation, itself dependent upon the conversion of an extracellular stimulus to an intracellular signal. In many model and pathogenic fungi, the integrity of a PM-associated complex of transmembrane proteins and cognate arrestins is essential for pH sensing; however, recent studies in *C. neoformans* have provided detailed examples of divergent sensing and signalling mechanisms. Although knowledge of how fungal pathogens sense environments has improved, there remains overt reliance upon understanding these mechanisms in model organisms. A selection of important unanswered questions is provided in Table 2.

**Table 2:** Unanswered questions on the pH adaptation mechanisms of model and pathogenic fungi

Species	Unanswered questions
All fungal pathogens	• Can pH signalling/sensing mechanisms be targets for novel antifungal discovery? • Is the response to alkalinisation ligand-mediated? • Which residues in the C-terminus of fungal pH sensors are critical for interaction with the PM? • Are the lipid entities governing fungal pH sensing conserved?
*A. fumigatus*	• What is the subcellular localization of the pH-sensing components? • What is the topology/structure of the pH sensing complex? • Is dissociation of the *Af*PalH C-terminus critical for pH sensing? • Does PalH form a multimeric pH sensor, as Rim21 does with Dfg16?
*C. albicans*	• How is ESCRT machinery, via *Ca*Vps23 recruited? • Is CaRim21 endocytosis essential for pH sensing? • Does Rim8 interact with downstream signalling components as in *A. nidulans* and *S. cerevisiae?*
*C. albicans, C. neoformans*	• What is the critical functional significance of conserved sensing attributes such as Vps23 and Snf7, in mechanistically divergent pathways?
*C. neoformans, C. albicans*	• How do complexes or pH sensors (*Cn*Rra1) become endocytosed?
*S. cerevisiae, C. neoformans*	• What drives the dissociation of the C-terminus of Rim21/Rra1? • Does dissociation occur when the full form of Rim21/Rra1 is expressed?
